# Representative *Bacillus* sp. AM1 from Gut Microbiota Harbor Versatile Molecular Pathways for Bisphenol A Biodegradation

**DOI:** 10.3390/ijms22094952

**Published:** 2021-05-07

**Authors:** Ana López-Moreno, Alfonso Torres-Sánchez, Inmaculada Acuña, Antonio Suárez, Margarita Aguilera

**Affiliations:** 1 Department of Microbiology, Faculty of Pharmacy, University of Granada, Campus of Cartuja, 18071 Granada, Spain; alopezm@ugr.es (A.L.-M.); alfons_ats@ugr.es (A.T.-S.); 2 Instituto de Nutrición y Tecnología de los Alimentos, INYTA-Granada, 18100 Granada, Spain; iacuna@ugr.es (I.A.); asuarez@ugr.es (A.S.); 3 Department of Biochemistry and Molecular Biology, Faculty of Pharmacy, University of Granada, Campus of Cartuja, 18071 Granada, Spain; 4 IBS: Instituto de Investigación Biosanitaria ibs., 18012 Granada, Spain

**Keywords:** human microbiota, *Bacillus*, bisphenols, molecular pathways, enzymes, EPS, PHA

## Abstract

Human gut microbiota harbors numerous microbial species with molecular enzymatic potential that impact on the eubiosis/dysbiosis and health/disease balances. Microbiota species isolation and description of their specific molecular features remain largely unexplored. In the present study, we focused on the cultivation and selection of species able to tolerate or biodegrade the endocrine disruptor bisphenol A (BPA), a xenobiotic extensively found in food plastic containers. Chemical xenobiotic addition methods for the directed isolation, culturing, Whole Genome Sequencing (WGS), phylogenomic identification, and specific gene-encoding searches have been applied to isolate microorganisms, assess their BPA metabolization potential, and describe encoded catabolic pathways. BPA-tolerant strains were isolated from 30% of infant fecal microbial culture libraries analyzed. Most isolated strains were phylogenetically related to the operational taxonomic group *Bacillus amyloliquefaciens* spp. Importantly, WGS analysis of microbial representative strain, *Bacillus* sp. AM1 identified the four complete molecular pathways involved on BPA degradation indicating its versatility and high potential to degrade BPA. Pathways for Exopolysaccharide (EPS) and Polyhydroxyalkanates (PHA) biopolymer synthesis were also identified and phenotypically confirmed by transmission electronic microscopy (TEM). These microbial biopolymers could generally contribute to capture and/or deposit xenobiotics.

## 1. Introduction

The microorganisms that colonize the human gut are functionally diverse and highly variable [1]. Four phyla, Firmicutes, Bacteroidetes, Actinobacteria, and Proteobacteria, dominate the human gut. Daily exposure to industrial xenobiotics found in dermal products, food, and beverage containers trigger alterations in gut microbiota composition [2]. Dysbiosis, an imbalance in the composition and metabolic capacity of our microbiota, has been related to the cumulative intake of a wide variety of xenobiotic substances that induce obesogenic phenotypes [3], and act as endocrine disruptors through microbiota interaction [4,5]. Bisphenol A (BPA) and its analogs (bisphenol-B, -S, -F and -E), used as building blocks of polycarbonate plastics, as well as parabens (methyl-, ethyl-, propyl- and phenyl-paraben), used as antimicrobial preservatives, have been associated with metabolic and endocrine/hormonal diseases [6,7]. Phthalates, such as di-(2-ethylhexyl) phthalate, are used as plasticizers and have been shown to exert obesogenic effects, affect the reproduction system, and modify key glycemic parameters [8,9]. Perchlorates, organophosphates [10], and other pesticides trigger symptoms of diabetes and obesity [11].

Numerous gut microorganisms belonging to the Firmicutes phylum possess a diverse and versatile array of enzymes. Species within the *Bacillus* genus are consistently identified in the human gut and are a source of enzymes with potential technological applications in the modern food sector [12]. The use of bacterial enzymes in the current food industry has gained increasing recognition in recent years as a result of the demand for new and better products [13]. In this sense, due to the increase in the human population and the growing need for products to satisfy the nutritional requirements of an increasingly demanding population, enzymes of bacterial origin are presented as among the most versatile alternatives in the current industrial context [14]. Commensal microorganisms isolated from human microbiota could fulfill the criteria of safety assessment and the status of Qualified Presumption of Safety (QPS) [15,16]. In this sense, most species within the *Bacillus* subtilis cluster are considered QPS [17] and are increasingly considered as feed additives [18].

Taxonomic classification of *Bacillus* species and the specific WGS description of their encoded biochemical pathways are needed to consider the food and feed safety of *Bacillus* strains [19]. Ecological and phenotypical heterogeneity within the *Bacillus* genus have rendered pure 16S rDNA-based taxonomy inefficient to discern between *Bacillus* species, even leading to misclassifications in the past [20]. Next-Generation Sequencing platforms and in silico tools have been developed to perform appropriate phylogenomic classifications and predict metabolic pathways involved in industrial and environmental processes, expanding the molecular knowledge of the enzymatic arsenal for BPA degradation in *Bacillus* species. Few microbial cultivation studies explored the potential BPA degrading capacity of *Bacillus* species for environmental bioremediation [21]. Recent approaches to assess the degradation pathways of BPA in *Bacillus* and other microorganisms have provided a new field of research to understand in the context of industrial and environmental biotechnology [22,23].

BPA is a dietary xenobiotic banned at specific levels. Because gut microbiota is a potential source for screening the capacities of tolerance and biodegradation capacities, particular microorganisms might provide endogenous bioremediation and could become detoxifying probiotics. Thus, the main objective of the present study was to describe, for the first time, BPA-tolerant microorganisms from the human gut and the specific pathways that allow them to behave as active metabolizers of this endocrine disruptor, possibly modulating its physiological impact. Culturing isolation strategies and WGS descriptions are used to find the specific BPA-degrading genes involved in the degradation pathways.

## 2. Results and Discussion

### 2.1. BPA-Tolerant Microorganisms Isolated from Human Microbiota: 16S RNA Partial Gene Identification and Phylogenetic Analysis

Our hypothesis was to seek human microbes capable of tolerating and metabolizing the endocrine disruptor BPA [4], the most important bisphenol in a large family of chemicals that are utilized to produce polycarbonates and epoxy resins and found in foods, drinks, and baby bottles. A comprehensive approach involved the culturing and isolation of gut microbial species in BPA-enriched complete or minimal media for further morphology, phylogenetical, biochemical, as well as WGS analyses was undertaken. It is an untested innovative strategy for a rational and functional search of potential next generation probiotics for biotechnological use in BPA detoxification. This study sets the basis for future linked omics and multidisciplinary studies aimed at the identification of new BPA-degrading microbes in the human gut and their association with the short and long-term daily intake of BPA.

**Figure 1:**
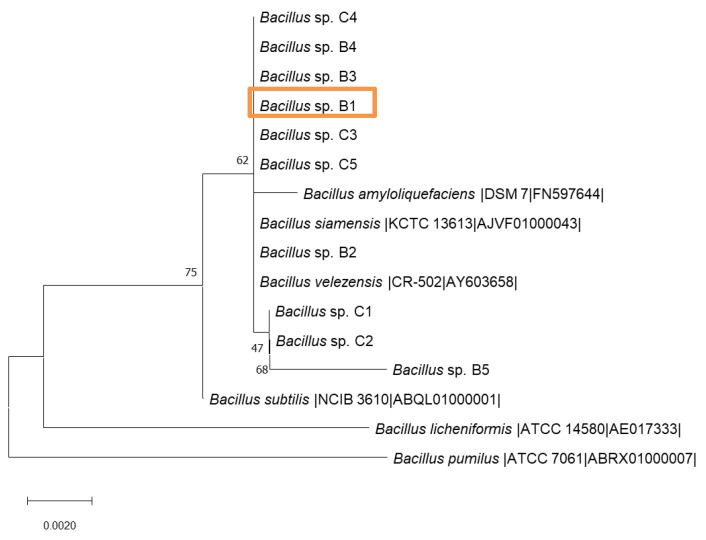
A phylogenomic tree based on partial 16S RNA gene (Table S1) of isolated strains of *Bacillus* sp. and closely related species. The tree was inferred by the maximum-likelihood method. The species and strain names are shown, and accession numbers are indicated in parentheses. Bootstrap values after 1000 resamplings are indicated at branch nodes. Bar: 0.0020 nucleotide substitution per site.

Isolation and identification of BPA-tolerant strains from microbiota samples were successful from the different concentrations used [0.5; 10; 20 and 50 ppm] and demonstrated a clear predominance of Firmicutes phylum taxa. This is one of the most populated phyla in microbiota with wide compositional, clinical, and functional variability [24,25]. Out of these 20 isolates analyzed, 83.3% were related to the genus *Bacillus*, and 16.7% of the strains were distributed within *Staphylococcus*, *Enterococcus*, and *Streptococcus* genera (Table S1). However, the analysis of these minority genera was beyond the objective of the present work. The phylogenetic tree based on partial 16S RNA gene of *Bacillus spp.* isolated strains showed a main cluster related to *B. amyloliquefaciens*, *B. velezensis* and *B. siamensis* (Figure 1), being only C1, C2 and B5 isolates different to the rest of *Bacillus spp.* The repeated presence of closely related *B. amyloliquefaciens* isolates in approximately 30% of the analyzed fecal samples suggested differential metabolic detoxifying potential of these species in individuals under high dietary exposure level to BPA [7] or food endocrine microbiota disruptors (EMD) [4].

Moreover, preliminary data on parallel metagenomics analyses were in concordance with this percentage of *Bacillus* sp. presence (data not shown). We hypothesize that the long-term daily intake of xenobiotic compounds such as BPA or parabens present in food containers may select specific taxa groups in the intestinal microbiota, thus increasing or reducing microbial populations according to their enzymatic capacity to tolerate and metabolize BPA. Increased isolation of species *Bacillus amyloliquefaciens* in individuals might reveal a clinical potential impact through high tolerance to BPA [26].

Interestingly, the potential BPA metabolization by *Bacillus* spp. has solely been investigated in relation to environmental and bioremediation resources [22,23]. The present study constitutes the first report on BPA-tolerant human microbiota species that might became a new extensive biotechnological source of probiotics for plants, food, and clinical purposes. In this sense, several *Bacillus* strains are proposed for being anti-obesogenic probiotics [27,28]. Still, the major limitation for using these new potential probiotics is the lack of standardized criteria and protocols for target disorders. Next-generation probiotics for obesity-related diseases promise to improve the rational of clinical trial design and outcomes [29]. Additional microbial food safety and risk assessments would be needed for strains proposed for food and feed, including strain characterization and genome sequencing, screening for undesirable attributes and metabolites, and experimental evidence of safety by appropriately designed safety evaluation studies [18,30].

### 2.2. Identification of the Representative BPA-Tolerant Microbiota Strain and Morphological Features

**Figure 2:**
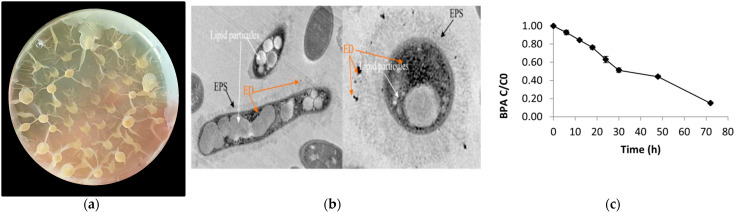
(**a**) Colony morphology of *Bacillus* sp. in MRS medium; (**b**) Image of *Bacillus* sp. AM1 by TEM showing lipidic PHA granules, EPS, and Endocrine disruptor ED-BPA. 1µm scale; (**c**) BPA degradation curve by *Bacillus* sp. AM1. BPA biodegradation capacity was tested in TSB liquid cultures of *Bacillus* sp. AM1 exposed to 25 ppb concentration of BPA at 30ºC for 72h (LC-MS/MS system for quantification).

All isolated and typified strains were able to tolerate between 0.5 and 50 ppm of BPA. *Bacillus* sp. AM1 (B1) was the BPA-tolerant selected strain representing the main phylogenetic cluster (Figure 1) for being further analyzed (phenotypic features and WGS). This selection was done based on the following enzymatic and phenotypic characteristics preliminary analyzed: the B1 strain was able to tolerate higher concentration of BPA; B1 showed special morphological and phenotypic features, such as biopolymers EPS and PHA production that can protect from the xenobiotic exposure (Figure 2), specific gene-encoding enzymes for both biopolymers were also found in the WGS of *Bacillus* sp. AM1 (Tables S2 and S3), and the phylogenetic identification as shown in Figure 1, B1 may represent the major close-related group. This cluster represented by the B1 strain and the grouping of B3, B4, C3, C4, and C5 strains was closely related to the operational *B. amyloliquefaciens* group [20], and became the most frequently identified related species from the microbiota samples analyzed.

*Bacillus* sp. AM1 is an anaerobic facultative rod with subterminal swollen spores. Pale yellow colonies forming oleaginous drops in MRS medium are typical (Figure 2a). Similarly, lipid, wax ester, or PHA producers showed similar morphologies and characteristics [31,32]. Transmission electron microscopy (TEM) revealed the presence of clear cytoplasmatic lipid accumulations similar to PHA granule accumulation. We also observed biopolymers type capsular EPS (Figure 2b).

The hydrolytic enzyme capacities of *Bacillus* sp. AM1 (Table 1) revealed similar results to those described for *Bacillus amyloliquefaciens*, *B. velezensis*, and *B. siamensis*, including inulinase activity. *Bacillus* genus is considered of interest for its enzyme productivity for many manufacturing processes [33,34,35]. The antibiotic sensitivity testing revealed that *Bacillus* sp. AM1 was resistant to ampicillin, ceftazidime, penicillin, and amoxicillin/clavulanic acid (Table 2). These resistances were previously described as intrinsic resistance showed commonly by *Bacillus* spp., not posing any safety concern [18].

*Bacillus* sp. AM1 was able to grow in BPA-enriched M9 medium (0.5 ppm) showing slower colony development compared to the control and M9 containing glucose as sole carbon source (0.5 ppm). Conversely, *Bacillus* sp. AM1 was not able to grow on M9 medium containing BPS (0.5 ppm), an analogue of BPA, indicating that BPS may be toxic or cannot be used as sole carbon source by this strain (Figure S1). *Bacillus* sp. AM1 was capable of degrading 84.68% of BPA after 72 h, using a starting BPA concentration of 25 ppb (Figure 2c). A similar pattern of biodegradation was shown by *Bacillus* sp. GZB [22].

### 2.3. Whole Genome Sequence of Bacillus sp. AM1 Analysis

**Figure 3:**
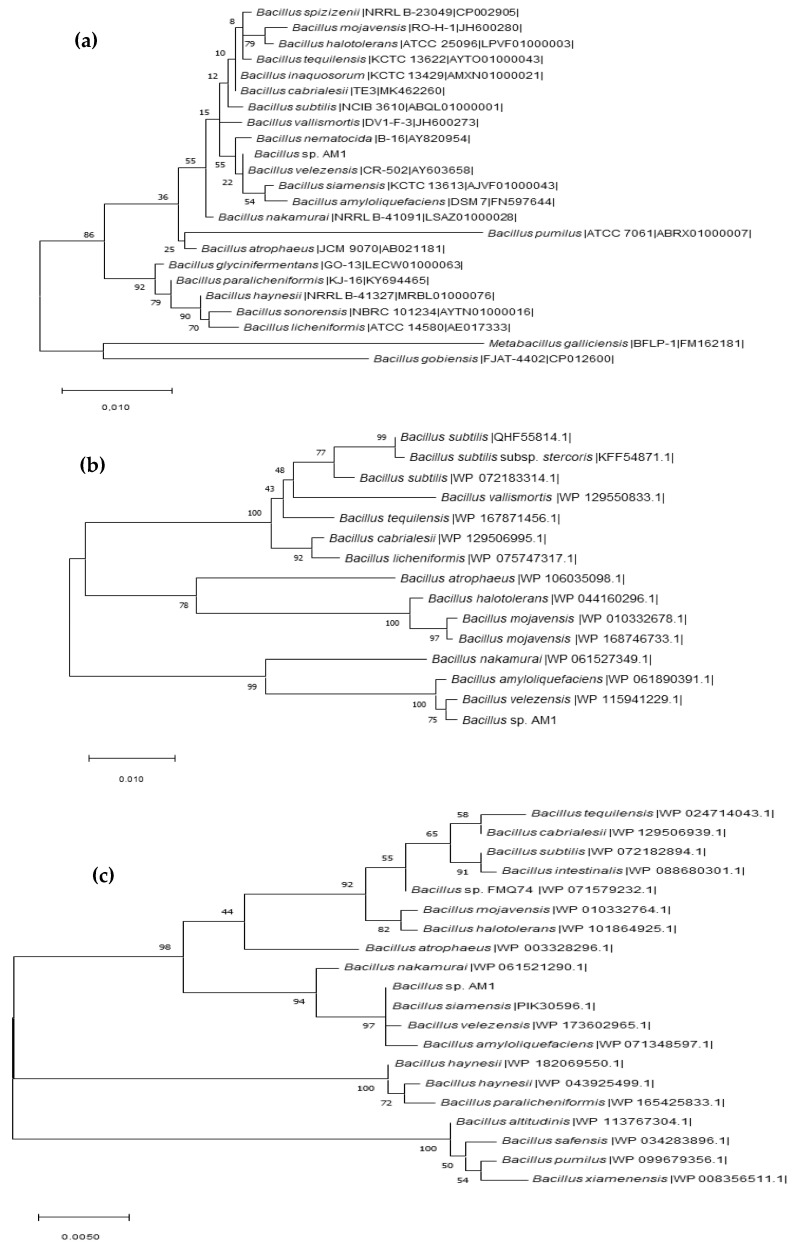
Phylogenomic trees based on gene sequences of *Bacillus* sp. AM1: (**a**) 16S RNA gene; (**b**) *gyr*A gene, similarity distances were computed using JTT’s model; (**c**) *rpo*B gene, similarity distances were computed using JTT’s model. The tree was inferred by the maximum-likelihood method. The species and strain names are shown, and accession numbers are indicated in parentheses. Bootstrap values shown after 1000 resamplings.

WGS of *Bacillus* sp. AM1 (GenBank CP047644.1) showed a total length (bp) of 4,203,926 and a G+C content value of 45.98 % (Figure S2). The genome of strain *Bacillus* sp. AM1 shared 67.67–98.36% ANI values with the *Bacillus subtilis* cluster (Table S4). DNA–DNA hybridization (isDDH) relatedness between strain *Bacillus* sp. AM1 and the known *Bacillus* species ranged from 28.2 to 83%. Results from the ANI and isDDH analyses and the phylogenomic trees based on 16S RNA gene (Figure 3a), *gyr*B (Figure 3b) and *rpo*B (Figure 3c) suggested that strain *Bacillus* sp. AM1 belongs to the safe operational group *B. amyloliquefaciens* [20], with no pathogenic or virulence factors or toxin genes. Moreover, rMLST analysis showed match to 55 genes and, within them, to 6 genes unique and specific of *Bacillus* sp. AM1: *rpm*D, *rpl*M, *rpl*I, *rpl*E, *rpl*A, *rps*G. We are aware that many species of *Bacillus* are difficult to be taxonomically classified under the universal rules; thus additional phylogenomic and phenotypic data will be obtained for a more precise classification. Moreover, EPS and PHA biosynthesis complete gene pathways (Tables S2 and S3) were also identified, confirming the features observed by TEM analyses (Figure 2b).

### 2.4. Description of Putative BPA Biodegradation Pathways by Microbiota BPA-Tolerant Isolate

**Figure 4:**
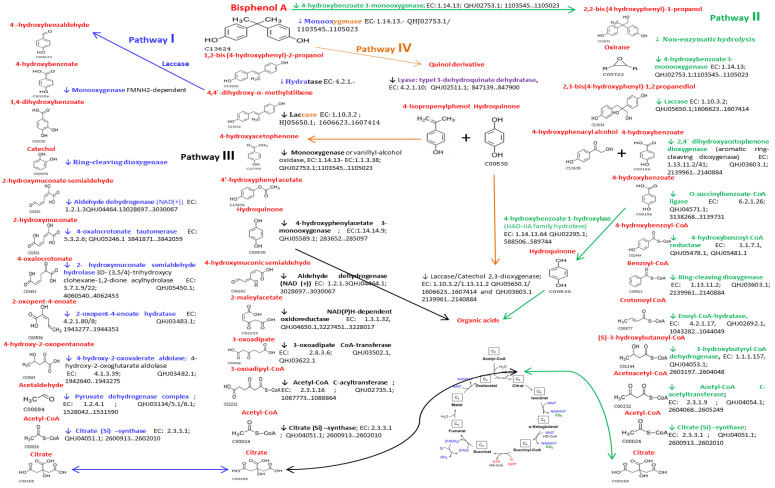
Proposed Biodegradation Pathways of BPA based on the WGS analysis of *Bacillus* sp. AM1: Reaction steps, Enzymes, EC number, protein ID, and specific genes loci.

BPA biodegradation occurs due to the contribution and coordination of many expressed protein-coding genes. Complete gene-encoded enzymes for the four reference pathways of BPA degradation were found in the *Bacillus* sp. AM1 genome, a major difference with *Bacillus* sp. GZB strain, isolated from recalcitrant environment, whose description seemed to lack 30% of these enzymes [22]. Description of reaction steps, specific enzymes, EC number, genes loci, and protein ID from each pathway are shown in Figure 4. This versatile gene arsenal for BPA metabolization enzymes should be confirmed with functional and chemical analyses, but underscores the huge potential of BPA biodegradation, possibly generating intermediate metabolites with more or less estrogenic-linked effects [22,36]. Our results suggest that the establishment of routes linked to the degradation of estrogenic xenobiotics, such as BPA, involves the participation of a large number of microbial enzymes whose joint activity entails the production of a multitude of intermediate less toxic byproducts which can even be found related to other metabolic routes or destined to the energy production through the Cycle of Tricarboxylic Acids [23]. Likewise, numerous studies related to the metabolism of BPA highlighted the central role of cytochrome P450 in bacteria [37,38].

Lobos et al. [39] and Sasaki et al. [40] showed the existence of microorganisms belonging to *Sphingomonas* and *Bacillus* genera capable of using BPA as a carbon source through its degradation in multiple stages, with the production of end byproducts of variable toxicity.

## 3. Materials and Methods

### 3.1. Tolerant-BPA bacterial Isolation from Human Gut Microbiota with Biotechnological Interest

Ten microbial isolates from fecal human microbiota collections of 0 to 1 year old infants (Isolates B- Project INFABIO) and 6–8-year-old children (Isolates C-Project OBEMIRISK) were obtained by a serial dilution method, exposition to different BPA concentrations [0.5, 10, 20, and 50 ppm] during 72 h and further spreading in BHI/MRS media incubated under aerobic and anaerobic conditions (anaerobic jars anaerocult^®^) at 37 °C.

The BPA-tolerant bacterial colonies with distinguishing features were isolated as pure culture for subsequent morphological, phenotypic and genotypic identifications: bacterial cell counts, gram staining, spore staining, capsule staining, catalase activity, oxidase, and motility tests.

### 3.2. Genomic DNA from Tolerant-BPA Bacterial Isolates, Taxonomy Identification and Phylogenetic Analysis

Genomic DNA was extracted using DNAeasy columns (Qiagen^®^, Germany) following the manufacturing instructions. The isolated DNA was quantified using Nanodrop (Thermo Scientific) and biophotometer (Eppendorf^®^ D30). The quality of DNA was monitored through gel electrophoreses. 16S RNA gene sequencing of selected bacterial strains was done by Sanger method (IPBLN Service). Forward and reverse sequences were provided separately. Reverse sequence was converted to complementary sequence with Chromas Pro 2.0 software (Technelysium Pty Ltd., Tewantin, Australia). Sequences were inspected for maximum homology against GenBank using NCBI’s BLASTn program. The collection of 16S RNA partial gene sequences is done using the Ezbiocloud platform. 

### 3.3. Morphological Features and Identification of BPA-Tolerant Bacterial Strains

The selection of a representative sample from fecal BPA-tolerant microorganisms for further phenotypic and WGS analysis was done according to specific phenotypic features and specific phylogenetic classification.

Transmission electron microscopy was used to examine possible structural changes in the selected strain. The samples were obtained after exposing the bacteria to the different BPA treatments. The techniques detailed below were carried out in the Laboratory for the Preparation of Biological Samples of the Microscopy Service of the Center for Scientific Instrumentation (CIC) of the University of Granada. Samples were fixed with 2.5% glutaraldehyde in sodium cacodylate buffer (pH7.4, 0.05M) for 24 h at 4 °C. After washing them three times in sodium cacodylate buffer (pH7.4, 0.1M) for 15 min, the post-fixation was carried out with 1% osmium tetroxide + 1% potassium ferricyanide in water for 2 h. It was washed again three times in water 15 min. And dehydration was realized using ascending ethanol gradients (50%, 70%, 90%, and 100%) for 15 min. Subsequently, the infiltration was carried out in a mixture of ethanol/resin (Epon 812) 2:1, 2:2 and 1:2 for 1 h and then it was the inclusion in pure resin overnight. After polymerization at 60 °C for 48 h, ultra-fine sections of 700 Angstrom were made (in a Leica Ultracut R ultramicrotome) deposited on copper grids and contrasted with uranyl acetate and lead citrate.

The enzymatic assay were also determinate for starch, carboxymethyl cellulose, gelatin, inulin, tween 80, DNase supplemented media were used to test several enzymes production according to complementary methodologies previously described [41,42].

The antimicrobial resistance patterns of *Bacillus* sp. AM1 to 17 clinically important antibiotics, included those antibiotics recommending on the EFSA guidelines for testing antimicrobial susceptibility of the *Bacillus* species [43], were conducted using the agar disc-diffusion method. The strains were characterized as sensitive or resistant based on the size of the inhibition zones around each disc, according to the National Committee for Clinical Laboratory Standards (CLSI) criteria [44].

### 3.4. BPA Tolerance Testing

BPA biodegradation capacity was tested in TSB liquid cultures of *Bacillus* sp. AM1 exposed to 25 ppb concentration of BPA at 30 °C during 72 h. Microbial preparations were measured through LC-MS/MS system for BPA quantification. Minimal medium 9 (M9) and M9 containing BPA (0.5 ppm) as sole carbon source were used for the isolation of BPA tolerant or biodegradation strain. M9 containing glucose (0.5 ppm) and M9 containing BPS (0.5 ppm) were also used to compare the growth ability. Chemicals, reagents, instrumentation and software for bisphenols determination were carried out by CIC services under validated procedures previously described by García-Córcoles et al. [45].

### 3.5. Whole Genome Sequence of BPA-Tolerant Bacterial Strain

Genomic DNA extraction for WGS: *Bacillus* sp. AM1 (B1) selected for its phenotypic characteristics and its potential in biotechnology was inoculated in 2 mL nutrient broth, followed by overnight incubation at 37 °C. The cells were harvested by centrifugation at 5000× *g* for 10 min. Genomic DNA was extracted using DNAeasy columns (Qiagen^®^, Germany) as previously described.

High-throughput sequencing: Shotgun library preparation of *Bacillus* sp. AM1 (B1) strain was performed using the Nextera DNA Sample Preparation kit (Illumina, USA). Clusters generation and the sequencing were performed using the TruSeq™ PE Cluster kit v2-cBot-HS and TruSeq SBS v3-HS (200 cycle) kit respectively (Ilumina, USA). The sequencing was performed on the HiScan SQ sequencer (Illumina, USA).

Genome assembly and annotation: Hybrid de novo assembly was performed with Illumina short reads (c.a. 440X coverage, 150bp pair end reads) and Oxford Nanopore long reads (c.a. 1800X coverage) using Unicycler1 with the “conservative” option and threads 10 parameter [46].

### 3.6. Whole Genome Sequence Analyses

#### 3.6.1. Phylogenomic Analysis

Genome-based taxonomic classification and genome assembly were searched at Microbial Genomes Atlas (MiGA) online server (http://www.microbial-genomes.org accessed on 20 February 2021) that uses NCBI non-redundant prokaryotic genomes database, JSpeciesWS (http://jspecies.ribohost.com/jspeciesws accessed on 20 February 2021), and TrueBacTM ID system from the EzBioCloud server (https://www.ezbiocloud.net/contents/genome accessed on 20 February 2021). The species identification carried out in TrueBac-ID was based on the comparison of average nucleotide sequence identity (ANI) between the genomes of *Bacillus* sp. AM1 and the related type strains. For a reliable identification of the strains, the Type (Strain) Genome Server (TYGS) was employed. Prediction from identified Ribosomal Multilocus Sequence Typing (rMLST) alleles linked to genomes (53 rps genes) was used as a means of integrating microbial taxonomy and typing [47]. 16S RNA gene and the housekeeping genes gyrB and rpoB sequences were used for phylogenomic identification.

Prokaryotic genome comparisons were performed by ANI calculator using the OrthoANIu algorithm available on Ezbiocloud [48]. Digital or isDDH analyses were carried out by Genome-to-Genome Distance Calculator (GGDC) (formula 2) [49].

#### 3.6.2. Molecular and Gene Prediction and Annotations

Draft *Bacillus* sp. AM1 genome was annotated using the NCBI Prokaryotic Genome Annotation Pipeline (PGAP). The functional annotation of genes was also carried out using web-based Rapid Annotation Using Subsystem Technology (RAST) annotation server (http://rast.theseed.org/FIG/rast.cgi accessed on 20 February 2021). Protein coding genes were scanned for their organization into operons using the web server Operon-mapper. Functional annotation of genes in terms of Kyoto Encyclopedia of Genes and Genomes (KEGG) orthology assignments and predictions of KEGG pathways were carried out through KEGG Automatic Annotation Server (KAAS) server (https://www.genome.jp/kegg/kaas/ accessed on 20 February 2021) using bi-directional best hit (BBH) method in GhostZ. Similarly, the *Bacillus* sp. AM1 genome were also scanned for cluster of orthologous groups (COGs) annotations using eggNOG-mapper v2 (http://eggnog-mapper.embl.de accessed on 20 February 2021). Automated Carbohydrate-active enzyme ANnotation web server, (dbCAN meta server (http://bcb.unl.edu/dbCAN2/blast.php accessed on 20 February 2021), was employed to annotate Carbohydrate-Active enZYmes (CAZy).

#### 3.6.3. Identification of Microbial Enzymes Isolated from Bisphenol A Tolerant Microorganisms

Specific genes and amino acid sequences of the BPA-tolerant representative microorganism were searched by screening according to phenotypic and chemical characteristics such as EPS, PHA, and other enzymes related to the BPA degradation pathways: quinol-enzymes, laccase, through BLAST (NCBI), EZBIOCLOUD, BRENDA, UNIPROT, and EXPASY. A more exhaustive and combined analysis using the KEGG database (Kyoto Encyclopedia of Genes and Genomes) allowed corroborating the identity of the enzymes involved in metabolic pathways of BPA degradation previously describe by Li et al. [22]. EC numbers results essential for the screening in WGS *Bacillus* sp. AM1 protein encoding genes.

## 4. Conclusions

This study is the first report on the isolation of BPA-degrading microbes from the human gut and its complete description of BPA biodegradation pathways. The isolation of strains belonging to the Firmicutes phyla from human microbiota able to biodegrade BPA was successful through a BPA-enriched media and a directed cultivation approach. WGS analyses of representative *Bacillus* sp. AM1 allowed taxonomic and phylogenomic identifications, relevant phenotypic features confirmation, and safety assessment aspects. *Bacillus* sp. AM1 WGS revealed the presence of a complete arsenal of genes that encode enzymes for versatile BPA degradation; this might open avenues for the investigation of the use of these strains for environment, feed and food industries, and/or to explore the clinical modulation impact on endocrine pathogenesis modulation by BPA-degrader species.

## Figures and Tables

**Table 1:** Results derived from the enzymatic analysis of *Bacillus* sp. AM1.

Enzyme Test	Microorganisms					
	*Bacillus* sp. AM1	*B. amyloliquefaciens* (DSM7)	*B. velezensis* (CR-502)	*B. siamensis* (KCTC13613)	*B.cereus (B7)*	*B.mycoides* *(B12)*
Starch	+	+	+	++	++	++
Carboxymethylcelulose	−	−	−	−	−	−
Gelatine	−	−	−	−	−	−
Inuline	+	−	−	−	+	+
Tween 80	−	+	+	−	−	−
DNase	++	+	+	−	−	−

**Table 2:** Antibiotic sensitivity testing.

Antibiotic	Concentration (μg)	Resistance (R) Sensitive (S)	Zone inhibition (mm)
Ampicillin	10	R	-
Amikacin	30	S	14
Ceftazidime	30	R	-
Ciprofloxacin	5	S	40
Chloramphenicol	30	S	42
Clindamycin	2	S	22
Erythromycin	15	S	25
Gentamicin	10	S	22
Imipenem	10	S	50
Penicillin	10	R	-
Rifampicin	30	S	29
Vancomycin	30	S	22
Kanamycin	30	S	19
Streptomycin	10	R	-
Tetracycline	30	S	17
Co-Trimoxazole	25	S	39
Amoxicillin/ Clavulanic acid	20/10	R	-

## Supplementary Materials

The following are available online at https://www.mdpi.com/article/10.3390/ijms22094952/s1.

## Patents

IPR-823 Application in progress.
