# Supplementary material for: Representative Bacillus sp. AM1 from Gut Microbiota Harbor Versatile Molecular Pathways for Bisphenol A Biodegradation
Source: Int J Mol Sci. 2021 May 7;22(9):4952. doi: 10.3390/ijms22094952 (PMC8125285; doi:10.3390/ijms22094952)
Supplement: Supplementary file 1 [file ijms-22-04952-s001.zip › ijms-1121903-SI.pdf]

Supplementary Material I

Table S1. Isolates from human microbiota samples and 16S RNA partial gene homology description.

Microbiota Isolates	Best hit	bp position 16S RNA gene	Query cover (%)	Identity (%)	Accession Number
C1	*Bacillus siamensis* [LRM10-3D]	430 - 1150	100	100	MT645306.1
	Bacillus velezensis [XC1]		100	100	MT649755.1
C2	*Bacillus siamensis* [LRM10-3D]	430 - 1150	100	100	MT645306.1
	Bacillus velezensis [XC1]		100	100	MT649755.1
C3	*Bacillus amyloliquefaciens* [SRCM 112835]	430 - 1150	100	100	MT626036.1
	Bacillus velezensis [XC1]		100	100	MT649755.1
C4	*Bacillus* sp. [hb97]	430 - 1150	100	100	KF863885.1
	Bacillus siamensis [Van19-4]		100	99.86	JX065213.1
	Bacillus amyloliquefaciens [AD8]		100	99.86	MW325953.1
C5	*Bacillus amyloliquefaciens* [SRCM 112835]	430 - 1150	100	100	MT626036.1
	Bacillus siamensis [LRM10-3D]		100	100	MT645306.1
	Bacillus velezensis [XC1]		100	100	MT649755.1
C6	*Uncultured organism*	430 - 1150	100	100	HQ745049.1
	Enterococcus faecalis [2675]		100	99.87	MT611694.1
	Enterococcus faecalis [2674]		100	99.87	MT611693.1
B1	*Bacillus subtilis* [J-5]	430 - 1150	100	99.80	CP018295.1
	Bacillus amyloliquefaciens [UMAF6614]		100	99.87	CP006960.1
	Bacillus velezensis		100	99.87	MK092976.1
B2	*Bacillus amyloliquefaciens* [SRCM 112835]	430 - 1150	100	100	MT626036.1
	Bacillus velezensis [XC1]		100	100	MT649755.1
	Bacillus siamensis [LRM10-3D]		100	100	MT645306.1
B3	*Bacillus amyloliquefaciens* [SRCM 112835]	430 - 1150	100	99.86	MT626036.1
	Bacillus velezensis [XC1]		100	99.86	MT649755.1
	Bacillus siamensis [LRM10-3D]		100	99.86	MT645306.1
B4	*Bacillus amyloliquefaciens* [SRCM 112835]	430 - 1150	100	99.86	MT626036.1
	Bacillus velezensis [XC1]		100	99.86	MT649755.1
	Bacillus siamensis [LRM10-3D]		100	99.86	MT645306.1
B5	*Bacillus amyloliquefaciens* [MG3047]	430 - 1150	100	99.72	MW433897.1
	Bacillus velezensis [XC1]		99	100	MT649755.1
	Bacillus siamensis [IA1]		99	100	MW361063.1
B6	*Streptococcus salivarius* [2789]	430 - 1150	100	100	MT611793.1
	Streptococcus salivarius [2658]		100	100	MT611678.1
	Streptococcus salivarius [2619]		100	100	MT611641.1
B7	*Staphylococcus pasteuri* [RPS6]	430 - 1150	100	100	MT539733.1
	Staphylococcus pasteuri [IsprtNis008]		100	100	MT500567.1
	Staphylococcus pasteuri [CT1]		100	100	MT072161.1

Table S2. Gene-encoding and corresponding enzymes involved in EPS biosynthesis.

Gene	Enzyme	Enzyme description	EC number	Gene locus CDS Protein ID	Reactions
*eps*O	EpsO	Pyruvyl transferase	EC: 2.-.-.-	3526057..3527022 QHJ04937.1	Transferase activity
*eps*N	EpsN	Aminotransferase class I/II-fold pyridoxal phosphate-dependent enzyme	EC: 2.6.1.-	3527001..3528173 QHJ04938.1	Transaminase activity
*eps*M	EpsM	Acetyltransferase	EC: 2.3.1.-	3528178..3528825 QHJ04939.1	Transferase activity
*eps*L	EpsL	Sugar transferase	EC: 2.-.-.-	3528822..3529430 QHJ04940.1	Transferase activity
*eps*K	EpsK	MATE family efflux transporter	-	1130278..1131645 QHJ02774.1	Integral membrane component
*eps*J	EpsJ	Glycosyltransferase	EC: 2.4.-.-	3530941..3531975 QHJ04942.1	Transferase activity
*eps*I	EpsI	Pyruvyl transferase	EC: 2.-.-.-	3531972..3533048 QHJ04943.1	Transferase activity
*eps*H	EpsH	Glycosyltransferase family 2 protein	EC: 2.4.-.-	3533053..3534090 QHJ04944.1	Transferase activity
*eps*G	EpsG	EpsG family protein	-	3534109..3535212 QHJ04945.1	Integral membrane component O4 family O-antigen polymerase
*eps*F	EpsF	Glycosyltransferase family 1 protein	EC: 2.4.-.-	3535216..3536352 QHJ04946.1	Transferase activity
*eps*E	EpsE	Glycosyltransferase	EC: 2.4.-.-	3536345..3537187 QHJ04947.1	Transferase activity
*eps*D	EpsD	Glycosyltransferase family 4 protein	EC: 2.4.-.-	3537184..3538323 QHJ04948.1	Transferase activity

Table S3. Proposed gene-encoding and corresponding enzymes involved in PHA biosynthesis.

Gene	Enzyme	Enzyme description	EC number	Gene locus CDS Protein ID	Reactions
phaA	PhaA	Acetyl-CoA C-acetyltransferase	EC:2.3.1.9	3374301..3375476 QHJ04794.1	2 Acetyl-CoA <=> CoA + Acetoacetyl-CoA
*pha*B	PhaB	3-oxoacyl-[acyl-carrier-protein] reductase	EC:1.1.1.100 EC:1.1.1.36	1662062..1662802 QHJ03260.1	Acetoacetyl-CoA reduction =>R-3-hydroxybutyriyl-CoA
*pha*C	PhaC*	Polymerase	EC:2.3.1.-	-	Polymerization
*pha*R	PhaR	enoyl-CoA hydratase/isomerase family protein / 3-hydroxyacyl-CoA dehydrogenase	EC:4.2.1.17	3375487..3377856 QHJ04795.1	3-hydroxybutyryl-CoA dehydrogenase Fatty acid metabolism
*pha*J	PhaJ	enoyl-CoA hydratase	EC:4.2.1.17	2914743..2915522 QHJ04362.1	Crosspathway with Fatty acid metabolism/B-oxidation

Table S4. WGS *Bacillus* sp. AM1 comparisons (Genbank Accession no:CP0476444.1)

Microorganism Species	Accession no	OrthoANI (%)	isDDH (%)	G+C content (%)	Total length (pb)
Bacillus amyloliquefaciens HM618	NZ_CP029466.1	98.36	83	46.28	4,021,851
Bacillus velezensis AGVL-005	CP024922.1	98.07	79.8	45.98	4,146,154
Bacillus siamensis SCSIO 05746	NZ_CP025001.1	94.32	54.5	45.99	4,268,316
Bacillus subtilis subtilis168	NZ_CP053102.1	77.59	18.7	43.25	4,316,079
Bacillus licheniformis DSM 13	NC_006270.3	72.73	17.3	46.19	4,222,645
Bacillus pumilus ASM299836V1	NZ_PVQT01000001.1	70.65	17.2	41.57	3,787,586
Bacillus coagulans DSM2314	NZ_CP033687.1	68.72	34.3	44.24	3,628,651
Bacillus clausii KSM-K16	NC_006582.1	67.47	28.2	44.75	4,303,871

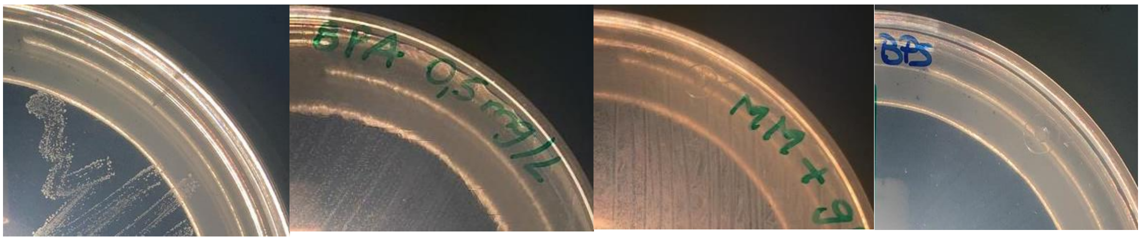

(**a**) (**b**) (**c**) (**d**)

Figure S1. Image of the growth of *Bacillus* sp. AM1 in diferent medias; (**a**) control bacteria growth in M9 without exposure to BPA; (**b**) bacteria growth in M9+BPA (0.5 ppm); (**c**) bacteria growth in M9+glucose (0.5 ppm); (**d**) bacteria growth in M9+BPS (0.5 ppm).

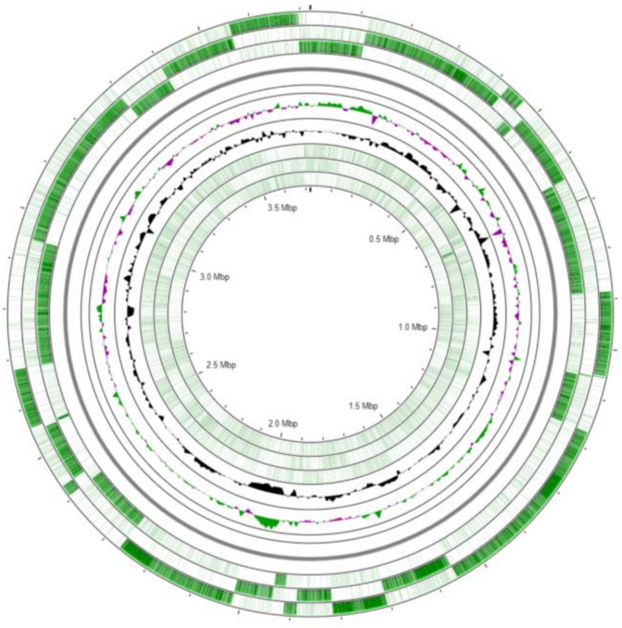

Figure S2. Circular whole genome secuence map of strain *Bacillus* sp AM1. Genome-map was drawn using Circos v0.64(http://circos.ca/).
